# Hypoxic preconditioning enhances mesenchymal stromal cell lung repair capacity

**DOI:** 10.1186/s13287-015-0120-3

**Published:** 2015-07-14

**Authors:** Fernanda Ferreira Cruz, Patricia Rieken Macedo Rocco

**Affiliations:** Laboratory of Pulmonary Investigation, Carlos Chagas Filho Biophysics Institute, Federal University of Rio de Janeiro, Centro de Ciências da Saúde, Avenida Carlos Chagas Filho, s/n, Bloco G-014, Ilha do Fundão 21941-902, Rio de Janeiro, RJ Brazil

## Abstract

Idiopathic pulmonary fibrosis is a progressive, irreversible, debilitating, and fatal lung disease, characterized by parenchymal fibrosis with reduced lung volumes and respiratory failure. No lasting option for therapy is available other than transplantation. Mesenchymal stem/stromal cells home to sites of injury, decrease inflammation, have antifibrotic properties, and promote epithelial tissue repair, so their use has been suggested as potential therapy for idiopathic pulmonary fibrosis. Despite reported benefits, the amount of mesenchymal stromal cells engrafting to the lung decreases substantially soon after administration. New strategies, such as hypoxia preconditioning, have thus been investigated in an attempt to optimize the engraftment, survival, and paracrine properties of stem cells. Hypoxia induces the expression of prosurvival mediators, chemoattractants, and growth factors involved in cell proliferation, migration, angiogenesis, antioxidant, antiapoptotic, and antifibrotic properties in mesenchymal stromal cells, optimizing their lung repair capability in an animal model of idiopathic pulmonary fibrosis.

See related research by Lan et al., http://www.stemcellres.com/content/6/1/97

## Introduction

Idiopathic pulmonary fibrosis (IPF) is a chronic, diffuse, fibrotic disease of the lung parenchyma of unknown etiology. Progressive deposition of collagen fibers in the lung interstitium inevitably leads to fatigue, dyspnea, hypoxia, and respiratory failure in patients with end-stage IPF. Although current pharmacological and nonpharmacological therapies can temporarily lessen the severity of symptoms, no effective treatment for IPF has been developed to rescue the impaired lung, prevent lesion progression, or both. Lung transplantation is considered the only curative approach. However, the significant shortage of suitable donor lungs and the many complications related to post-transplantation immunosuppression mean there is a dire need for new therapeutic approaches. In this context, mesenchymal stem/stromal cell (MSC)-based therapy is a promising alternative for the treatment of lung diseases. Novel strategies to optimize the beneficial effects of cell therapy have been investigated, seeking to enhance the proliferation, survival, engraftment, and paracrine properties of MSCs.

In a recent issue of *Stem Cell Research & Therapy*, Lan et al. [1] demonstrated that transplantation of hypoxia-preconditioned MSCs exerted better therapeutic effects in a mouse model of bleomycin-induced pulmonary fibrosis and enhanced the survival rate of engrafted MSCs, partially due to upregulation of hepatocyte growth factor (HGF).

## Main text

Repair of fibrotic lung parenchyma remains a significant clinical challenge. All currently available therapeutic trials in IPF are severely limited due to the lack of a clear understanding of the natural history of the disease. To date, treatment of IPF has been based on the concept that inflammation leads to injury; thus, most therapies have been based on eliminating or suppressing the inflammatory component through administration of anti-inflammatory agents including corticosteroids, immunosuppressants/cytotoxic drugs (e.g., azathioprine, cyclophosphamide), and anti-fibrotic agents (e.g., colchicine or D-penicillamine), alone or in combination [2]. Despite this variety of pharmacological options, no drug-based therapy has been proven unequivocally to alter or reverse the fibrotic process.

MSC-based therapy is an attractive alternative approach for the treatment of IPF, as MSCs home to sites of injury, inhibit inflammation, and contribute to epithelial tissue repair [3]. In addition, MSCs secrete paracrine mediators with antiapoptotic, anti-inflammatory, and antifibrotic effects [3]. Álvarez et al. [4] showed that lung-engrafted MSCs possessed the ability to ameliorate fibrotic effects in mice challenged with bleomycin. However, despite reported successes, the amount of engrafted MSCs decreased dramatically 1 day after transplantation due to exposure to harsh, toxic, and oxidative microenvironments [3]. New strategies to optimize cell therapy outcomes are therefore being investigated. A recent study involving overexpression of antioxidants, chemokine receptors, antiapoptotic genes, prosurvival genes, or growth factors in engrafted stem cells has shown improved cell survival following transplantation [5]. Additionally, cells may be preconditioned by sublethal exposure to selected stressors to induce prior expression of cytoprotective genes before subsequent lethal challenges [6]. Cellular preconditioning may include exposure of cells to physiological stimuli such as heat shock, small-molecule pharmacological agents, cytokines, growth factors, biophysical stimuli, or hypoxia [7]. Choosing appropriate preconditioning strategies may provide a simple yet effective way of promoting survival, enhancing regenerative properties, and boosting the tissue repair capability of transplanted cells in stem cell-based therapy [8].

The main insight of Lan et al.’s study was the use of hypoxic preconditioning (HP), which upregulated cytoprotective and regenerative genes, stabilized mitochondrial membrane potentials, increased homing ability, promoted cell proliferation, and acted against hydrogen peroxide-induced cell death in the treated MSCs. Moreover, hypoxia-preconditioned mesenchymal stem/stromal cells (HP-MSCs) attenuated bleomycin-induced cell apoptosis and extracellular matrix (ECM) production through transforming growth factor (TGF)-β1-mediated Akt signaling via paracrine effects. Lan et al. were the first to show that engraftment of HP-MSCs had therapeutic effects superior to those of untreated MSCs in a model of bleomycin-induced pulmonary fibrosis. Engafted HP-MSCs improved lung function, reduced lung edema, and reduced levels of proinflammatory and fibrotic factors. HP and stem cell transplantation have been extensively studied in a number of organs and tissues as a means of enhancing therapeutic effects in diseases such as stroke, myocardial infarction, traumatic brain injury, diabetes mellitus, inflammatory bowel disease, and acute kidney and liver injuries [7–9]. However, there have been no published studies on the use of HP-MSCs to treat pulmonary fibrosis.

Furthermore, Lan et al. demonstrated that transplantation of HP-MSCs attenuated ECM deposition, in a mechanism attributed to upregulation of HGF in the fibrotic lung. In this process, HGF plays a key role in preventing fibrosis or scar formation after injury, by inhibiting TGF-β-mediated myofibroblast differentiation and ECM production. HGF also exerts multiple protective effects on injured tissues via mitogenic, antiapoptotic, anti-inflammatory, and antifibrogenic signaling [10].

## Conclusion

Lan et al. [1] suggest that hypoxia-preconditioned MSC therapy enhanced the survival rate of engrafted MSCs, exerted superior therapeutic effects, and improved lung functions in bleomycin-induced pulmonary fibrosis in mice. Further studies should be conducted to ascertain whether HP-MSCs have similar beneficial effects in other models of fibrosis.

## Abbreviations

ECM: Extracellular matrix
HGF: Hepatocyte growth factor
HP: Hypoxic preconditioning
HP-MSC: Hypoxia-preconditioned mesenchymal stem/stromal cell
IPF: Idiopathic pulmonary fibrosis
MSC: Mesenchymal stem/stromal cell
TGF: Transforming growth factor
